# *Candida albicans* enhances meropenem tolerance of *Pseudomonas aeruginosa* in a dual-species biofilm

**DOI:** 10.1093/jac/dkz514

**Published:** 2019-12-22

**Authors:** Farhana Alam, Dominic Catlow, Alessandro Di Maio, Jessica M A Blair, Rebecca A Hall

**Affiliations:** 1 Institute of Microbiology and Infection, School of Biosciences, University of Birmingham, Edgbaston, Birmingham B15 2TT, UK; 2 Institute of Microbiology and Infection, College of Medical and Dental Sciences, University of Birmingham, Edgbaston, Birmingham B15 2TT, UK; 3 Birmingham Advanced Light Microscopy, School of Biosciences, University of Birmingham, Edgbaston, Birmingham B15 2TT, UK; 4 Kent Fungal Group, School of Biosciences, University of Kent, Canterbury, CT2 7NZ, UK

## Abstract

**Background:**

*Pseudomonas aeruginosa* is an opportunistic bacterium that infects the airways of cystic fibrosis patients, surfaces of surgical and burn wounds, and indwelling medical devices. Patients are prone to secondary fungal infections, with *Candida albicans* being commonly co-isolated with *P. aeruginosa*. Both *P. aeruginosa* and *C. albicans* are able to form extensive biofilms on the surfaces of mucosa and medical devices.

**Objectives:**

To determine whether the presence of *C. albicans* enhances antibiotic tolerance of *P. aeruginosa* in a dual-species biofilm.

**Methods:**

Single- and dual-species biofilms were established in microtitre plates and the survival of each species was measured following treatment with clinically relevant antibiotics. Scanning electron microscopy and confocal microscopy were used to visualize biofilm structure.

**Results:**

*C. albicans* enhances *P. aeruginosa* biofilm tolerance to meropenem at the clinically relevant concentration of 5 mg/L. This effect is specific to biofilm cultures and is dependent upon *C. albicans* extracellular matrix polysaccharides, mannan and glucan, with *C. albicans* cells deficient in glycosylation structures not enhancing *P. aeruginosa* tolerance to meropenem.

**Conclusions:**

We propose that fungal mannan and glucan secreted into the extracellular matrix of *P. aeruginosa*/*C. albicans* dual-species biofilms play a central role in enhancing *P. aeruginosa* tolerance to meropenem, which has direct implications for the treatment of coinfected patients.

## Introduction

The majority of infections in humans are polymicrobial in nature, with common diseases no longer considered to be caused by a single aetiological agent.^1^ The most prevalent polymicrobial infections include periodontitis, gastroenteritis, diabetic foot wounds, burn wounds and biofilm-associated infections.^1^^,^^2^

The genetic disease, cystic fibrosis (CF), is characterized by thickening of the mucus layer lining the endothelium of the respiratory tract, which provides an ideal environment for microbial colonization.^3^ Reduced mucociliary clearance enables these microorganisms to persist and form polymicrobial biofilms on the mucosa of the lower respiratory tract.^1^ The CF lung is a major site of interaction between *Pseudomonas aeruginosa* and *Candida albicans*.^4^^,^^5^ Around 70% of CF patients become chronically infected with *P. aeruginosa* by the age of 30,^6^ with *C. albicans* isolated from up to 75% of CF patients,^7^ although sputum samples are often contaminated with microbes from the upper respiratory tract and oral cavity.^8^ However, simultaneous colonization has been linked to severer clinical outcomes,^9^^,^^10^ due to accelerated decline in lung function and worsening of disease progression.^10^^,^^11^

Biofilms are structured communities of microbial cells ensnared within a matrix of extracellular polymeric substances.^12^^,^^13^ Biofilms are formed by bacterial and fungal species and an estimated 65%–80% of all microbial infections in humans are biofilm related.^2^^,^^14^ This has important clinical implications as the MICs of antimicrobials for biofilm cells can be 100–1000 times greater than for planktonic cells.^15^^,^^16^ Antimicrobial resistance in microorganisms poses an increasing challenge to public health worldwide,^17^^,^^18^ making biofilms a particularly relevant topic of research.

Previous work on interactions between *P. aeruginosa* and *C. albicans* has focused predominantly on physical and molecular interactions and their effects on growth, morphology and virulence.^19–24^ However, little is known of how their interactions affect antimicrobial drug efficacy. Studies on mono-species *C. albicans* and *P. aeruginosa* biofilms have linked biofilm extracellular matrix (ECM) material to antimicrobial drug inhibition. For example, the fungal polysaccharide β-1,3-glucan sequesters the antifungal fluconazole,^25^ whilst the *P. aeruginosa* exopolysaccharides Pel and Psl are implicated in the inhibition of various antibiotics, including tobramycin.^26^ Therefore, a greater understanding of the impact of this cross-kingdom interaction on antimicrobial tolerance is of great clinical importance.

Meropenem is a first-line antibiotic for treating *Pseudomonas* infections in the CF lung.^27^ Meropenem is a carbapenem β-lactam that targets PBPs within Gram-negative bacteria, causing inhibition of cell wall peptidoglycan synthesis, ultimately leading to osmotic lysis of bacterial cells.^28^^,^^29^ Meropenem is administered intravenously as a 15–30 min infusion of 1–2 g (adult dose), thrice daily for 2 weeks.^30^ When *P. aeruginosa* biofilms are treated with clinical doses of meropenem, only bacteria at the biofilm peripheries are killed, whilst cells closer to the base remain viable.^31^ In patients, the meropenem concentration found in epithelial lining fluid 1 h post-treatment is 5.3 mg/L,^30^ with the clinical breakpoint of *Pseudomonas* being >8 mg/L.^32^ Therefore, slight deviations in tolerance of *P. aeruginosa* to meropenem could impede clearance of the infection. Here, we observed that *P. aeruginosa*/*C. albicans* dual-species biofilms displayed enhanced tolerance to meropenem. This protection was provided through active secretion of fungal ECM components, specifically mannan and β-glucan. Therefore, co-colonization of *P. aeruginosa* and *C. albicans* within the CF lung may result in small reservoirs of protected *P. aeruginosa*, which could survive antimicrobial treatment and reseed the infection site.

## Materials and methods

### Strains and growth conditions

Strains of *P. aeruginosa* and *Candida* species used in this study are listed in Table 1. *P. aeruginosa* strains were maintained on, and cultured in, Miller-modified LB and *C. albicans* strains in yeast extract peptone dextrose (YPD) medium. Both were grown at 37°C, with aeration at 200 rpm. Antimicrobials (from Sigma–Aldrich, UK) were used at the following concentrations (mg/L): meropenem, 0, 1, 2.5, 5 and 10; ceftazidime, 0 and 5; ciprofloxacin, 0 and 0.05; tobramycin, 0 and 2; and fluconazole, 0, 250, 500, 750 and 1000.

**Table 1.** Bacterial and fungal strains used in this study

Strain	Common name	Genotype	Reference/source
*P. aeruginosa* strains			
ATCC 15692	PAO1	WT	ATCC
Midlands 1	Midlands 1	clinical isolate	^48^
*C. albicans* strains			
SC5314	SC5314	type strain	^56^
NGY152	NGY152	*ura3Δ::imm34/ura3Δ::imm434; RPS1/rps1Δ::URA3*	^57^
NGY355	*pmr1Δ*	*ura3Δ::imm434/ura3Δ::imm434; pmr1Δ::hisG/pmr1Δ::hisG; RPS10/rps10Δ::URA3*	^58^
NGY356	*pmr1Δ* + *PMR1*	*ura3Δ::imm434/ura3Δ::imm434; pmr1Δ::hisG/pmr1Δ::hisG; RPS1/rps1Δ::*CIp10*-PMR1*	^58^
CDH15	*mnn4Δ*	*ura3Δ::imm434/ura3Δ::imm434; mnn4Δ::hisG/mnn4Δ::hisG; RPS10::URA3*	^57^
CDH13	*mnn4Δ* + *MNN4*	*ura3Δ::imm434/ura3Δ::imm434; mnn4Δ::hisG/mnn4Δ::hisG; RPS10::* [CIp10*-MNN4-URA3*]_*n*_	^57^
NGY582	*mnn2Δ*	*ura3Δ::imm434/ura3Δ::imm434; mnn2Δ::dpi200/mnn2Δ::dpi200; RPS1/rps1Δ::*CIp10	^46^
NGY583	*mnn2Δ* + *MNN2*	*ura3Δ::imm434/ura3Δ::imm434; mnn2Δ::dpl200/mnn2Δ::dpl200; RPS1/rps1Δ::*CIp10-*MNN2*	^46^
NGY600	*Δmnn2–26*	*ura3Δ::imm434/ura3Δ::imm434; mnn2Δ::dpl200/mnn2Δ::dpl200; mnn22Δ::dpl200/mnn2Δ::dpl200; mnn23Δ::dpl200/mnn23Δ::dpl200; mnn24Δ::dpl200/mnn24Δ::dpl200; mnn26Δ::dpl200/mnn26Δ::dpl200; mnn21Δ::dpl200/mnn21Δ::dpl200; RPS1/rps1Δ::*CIp10	^46^
NGY337	*mnt1Δ*/*mnt2Δ*	*ura3Δ::imm434/ura3Δ::imm434; mnt1Δ::hisG/mnt1Δ::hisG; mnt2Δ:: hisG/mnt2Δ::hisG; RPS10/rps10Δ::*CIp10	^55^
NGY335	*mnt1Δ*/*mnt2Δ* + *MNT1*	*ura3Δ::imm434/ura3Δ::imm434; mnt1Δ::hisG/mnt1Δ::hisG; mnt2Δ:: hisG/mnt2Δ::hisG; RPS10/rps10Δ::*CIp10-*MNT1*	^55^
Non-*albicans Candida* strains			
WU284	*C. dubliniensis*	WT	^59^
CAY676	*C. tropicalis*	type strain	ATCC
CLIB214	*C. parapsilosis*	type strain	^60^
AM16/0701	*C. krusei*	clinical isolate	D. MacCallum, University of Aberdeen, Scotland, UK
ATCC 2001	*C. glabrata*	type strain	ATCC

### Formation of dual-species biofilms

Biofilms were grown in 96-well plates as previously described.^33^ Briefly, cultures were washed twice in PBS and *P. aeruginosa* cultures diluted to OD_600_ of 0.2 and *Candida* strains diluted to 1 × 10^6^ cells/mL in Mueller–Hinton broth (MHB) or DMEM supplemented with 1% l-glutamine. *P. aeruginosa* (2.4 × 10^6^) and *Candida* (1 × 10^5^) cells were incubated statically in flat-bottom 96-well plates for 2 h at 37°C to enable attachment, then non-adhered cells were removed and replaced with fresh medium. After 24h, the medium was replaced with fresh medium containing the appropriate amount of antimicrobial, or vehicle control, for an additional 18 h. To disrupt biofilms, medium was replaced with 100 μL of PBS containing 50 mg/L DNase and incubated at 37°C for 1 h. Biofilms were detached using a water bath sonicator, serially diluted 1 in 10 in PBS and plated onto cetrimide agar (to determine viable *P. aeruginosa* cfu) and YPD agar supplemented with 100 mg/L tetracycline (to determine viable *Candida* cfu). Experiments were performed with three technical and at least three biological replicates.

### Formation of P. aeruginosa biofilms in the presence of dead C. albicans or ECM components

To inactivate *C. albicans*, stationary-phase cultures were washed with PBS and cells either heat-killed at 100°C in PBS for 1 h or fixed in 1 mL of 4% paraformaldehyde (PFA) at room temperature for 1 h. Cells were then washed with PBS and diluted in MHB to 1 × 10^6^ cells/mL. Subsequently, biofilms were established and quantified as above.

Mono-species *P. aeruginosa* biofilms were established and grown for 24 h in MHB supplemented, at either 0 or 24 h, with 0.25 mg/mL glucan (from *Saccharomyces cerevisiae*), laminarin (from *Laminaria digitata*), mannan (from *S. cerevisiae*) or chitosan. All polysaccharides were obtained from Sigma–Aldrich, UK. After 24 h, medium was replaced with MHB containing the appropriate amount of antibiotic and biofilms were subsequently incubated and quantified as above.

### Scanning electron microscopy of biofilms

Biofilms were prepared for scanning electron microscopy using a previously published protocol^34^ with modifications. Single- and dual-species biofilms were grown on cell culture-treated plastic coverslips (Thermo Fisher Scientific) in 24-well plates for 24 h, after which the MHB medium was replaced with MHB with or without 5 mg/L meropenem. At 48 h, coverslips were washed twice with PBS and samples fixed in 2.5% glutaraldehyde in 0.1 M phosphate buffer for 1 h, at 4°C. Samples were dehydrated using increasing ethanol concentrations (50%, 70%, 90% and 100%) twice for 15 min each. Ethanol was replaced with liquid CO_2_ and heated up to the critical point to dry the samples. Each coverslip was mounted on a stub and sputter-coated with platinum. Scanning electron microscopy images were captured using a Philips XL30 ESEM-FEG environmental scanning electron microscope.

### Confocal microscopy of biofilms

Single- and dual-species biofilms with or without 5 mg/L meropenem were grown as above, scaled up to a final volume of 6 mL, in 6-well plates. Medium was replaced with PBS containing 5 mg/L propidium iodide (stains dead cells), 1 μM Syto 9 (dyes DNA) and 3 mg/L calcofluor white (stains fungal cell wall chitin) and incubated at 4°C in the dark for 1 h. Biofilms were then fixed by adding 4% PFA, incubated at 4°C in the dark for 1 h and then washed twice with PBS. Confocal microscopy was performed using a Leica SP8 system equipped with a Leica DM6 upright microscope, a ×40/0.80 objective and 402, 488 and 561 nm lasers. Biofilms were imaged directly in wells with a water-dipping lens. Z-stack scans were taken at two or three different areas within each well and processed with Fiji and LASx software.

### Planktonic assay

*P. aeruginosa* (2.4 × 10^7^) and *C. albicans* (1 × 10^6^) cells were added to 14 mL vent-capped culture tubes in a final volume of 2 mL MHB. Cultures were incubated for 3 h at 37°C with aeration at 200 rpm; the appropriate amount of antibiotic was added and cultures incubated for an additional 18 h. Cultures were sonicated in a water bath sonicator and serially diluted and plated for viable counts. Experiments were performed with three technical and four biological replicates.

### Determination of P. aeruginosa susceptibility to meropenem

MICs of meropenem were determined for *P. aeruginosa* strains according to the standardized broth microdilution method using MHB.^35^ Concentrations of meropenem used were 0, 1, 2, 4, 8, 16 and 32 mg/L. MICs were determined for *P. aeruginosa* strains using cells grown on LB agar and cells recovered from biofilms. MICs were the lowest concentrations of meropenem that caused no visible growth.

### Statistical analysis

Statistical analyses were done using GraphPad Prism 8.0.0 software. Data were analysed using two-way ANOVA and Holm–Sidak’s multiple comparisons test.

## Results

### C. albicans increases the tolerance of P. aeruginosa to meropenem in dual-species biofilms

**Figure 1.**
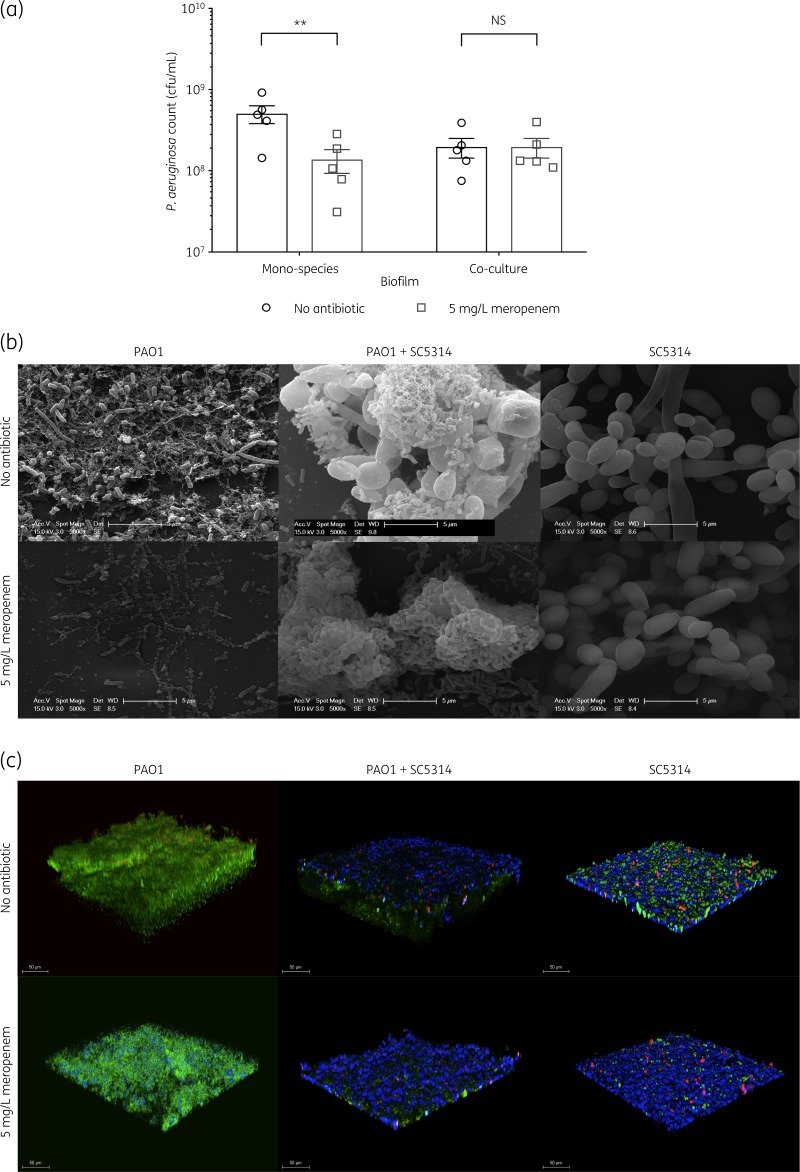
*C. albicans* increases the tolerance of *P. aeruginosa* to meropenem in a dual-species biofilm. (a) Preformed 24 h biofilms were incubated for 18 h in MHB containing no antibiotic or 5 mg/L meropenem. Data are the mean ± SEM from five biological replicates. Data were analysed using two-way ANOVA and Holm–Sidak’s multiple comparisons test (**P <* *0.01). NS, not significant. (b) Scanning electron microscopy analysis of biofilms. Meropenem treatment of mono-species *P. aeruginosa* biofilms results in death of bacterial cells, whilst the presence of *C. albicans* in the dual-species biofilm enhances meropenem tolerance; the tight association of *P. aeruginosa* cells to fungal surfaces is visible. *C. albicans* alone is unaffected by meropenem. (c) 3D reconstructions of biofilms from confocal z-stacks. Red indicates propidium iodide stain (dead cells), green indicates Syto 9 dye (DNA) and blue indicates calcofluor white stain (chitin).

To determine whether the presence of *C. albicans* within a *P. aeruginosa* biofilm can enhance tolerance of *P. aeruginosa* to meropenem, preformed mono-species (*P. aeruginosa*) and dual-species (*P. aeruginosa*/*C. albicans*) biofilms were treated with meropenem and *P. aeruginosa* survival quantified by viable counts. Given that the concentration of meropenem in the lung immediately after administration is between 5 and 6 mg/L,^30^ this drug concentration was the focus of the study. The viability of *P. aeruginosa* mono-species biofilms was reduced to 25.35% when treated with 5 mg/L meropenem, indicating *P. aeruginosa* biofilm cells are susceptible to meropenem. Fewer *P. aeruginosa* cells were recovered from dual-species biofilms, which is likely due to nutrient competition. However, in the presence of *C. albicans*, meropenem was non-effective against *P. aeruginosa* in both MHB (Figure 1a) and DMEM (Figure S1, available as Supplementary data at *JAC* Online), indicating that this inter–kingdom interaction negatively affects meropenem efficacy.

To visualize the structure of the biofilms, samples were analysed by scanning electron microscopy and confocal microscopy. Mono-species *P. aeruginosa* biofilms were significantly reduced in the presence of meropenem, while in the meropenem-treated dual-species biofilms, significant levels of *P. aeruginosa* colonized the fungal hyphae (Figure 1b), confirming the cfu data. In agreement with this, the biofilm thickness was lower in meropenem-treated dual-species biofilms (Figure 1c and Figure S2), indicating dense packing of bacterial cells against fungal hyphae, creating a more compact biofilm structure. Therefore, the presence of *C. albicans* enhances the tolerance of *P. aeruginosa* to meropenem.

To identify whether this dual-species interaction had any impact on antifungal resistance, biofilms were treated with fluconazole. *C. albicans* cells in dual-species biofilms showed similar susceptibility levels to fluconazole as untreated controls at all tested concentrations. Therefore, the presence of *P. aeruginosa* does not affect the antifungal activity of fluconazole under the tested conditions (Figure S3).

### Meropenem tolerance is not maintained following subculture of P. aeruginosa biofilm cells

The selective pressure from antibiotic use increases the likelihood of cells developing resistance. The sessile nature and close proximity of biofilm cells promotes cell–cell interactions,^36^ increasing horizontal gene transfer and mutation frequencies relative to planktonic cells.^37^ Furthermore, the presence of *C. albicans* increases *P. aeruginosa* mutation rates.^20^ To determine whether the observed increase in meropenem tolerance of *P. aeruginosa* was due to selection for resistance mutations, the meropenem MICs for cells recovered from both *P. aeruginosa* mono- and dual-species biofilms that had been treated with 5 mg/L meropenem, or untreated, were determined by standard broth microdilution MIC assay and compared with the MIC for the starter culture. The MIC for *P. aeruginosa* under all tested conditions was 4 mg/L, suggesting that *P. aeruginosa* cells recovered from treated biofilms were not resistant to meropenem and, therefore, the observed increased tolerance was unlikely due to selection for resistance mutations.

### Increased tolerance of P. aeruginosa to meropenem is specific to dual-species biofilms

Enhanced survival of *P. aeruginosa* as a result of interactions with *C. albicans* has previously been observed in planktonic cultures, through inter–kingdom communication via secreted metabolites.^21^ To determine whether the observed increase in meropenem tolerance of *P. aeruginosa* was specific to biofilms, *P. aeruginosa* susceptibility to meropenem in the presence of *C. albicans* was tested during planktonic growth. At all meropenem concentrations tested, there was no significant difference in *P. aeruginosa* survival whether in the presence or absence of *C. albicans* (Table 2), suggesting that *C. albicans*-mediated protection is biofilm specific.

**Table 2.** Increased *P. aeruginosa* meropenem tolerance in the presence of *C. albicans* is biofilm specific

Meropenem (mg/L)	Planktonic culture	*P. aeruginosa* count (cfu/mL), mean ± SEM	*P*
0	mono-species	4.00 × 10^9^ ± 4.14 × 10^8^	0.9040
	co-culture	3.81 × 10^9^ ± 2.78 × 10^8^	
1	mono-species	2.19 × 10^9^ ± 1.63 × 10^8^	0.3218
	co-culture	1.64 × 10^9^ ± 2.97 × 10^8^	
2.5	mono-species	7.36 × 10^8^ ± 8.39 × 10^7^	0.9650
	co-culture	8.12 × 10^8^ ± 1.57 × 10^8^	
5	mono-species	8.75 × 10^7^ ± 2.82 × 10^7^	0.9650
	co-culture	1.56 × 10^8^ ± 4.92 × 10^7^	

Planktonic cultures were grown for 3 h and subsequently incubated for 18 h in MHB containing 0, 1, 2.5 or 5 mg/L meropenem. Data are the mean ± SEM from four biological replicates. Data were analysed using two-way ANOVA and Holm–Sidak’s multiple comparisons test.

### Increased P. aeruginosa tolerance to meropenem is dependent on fungal viability

**Figure 2.**
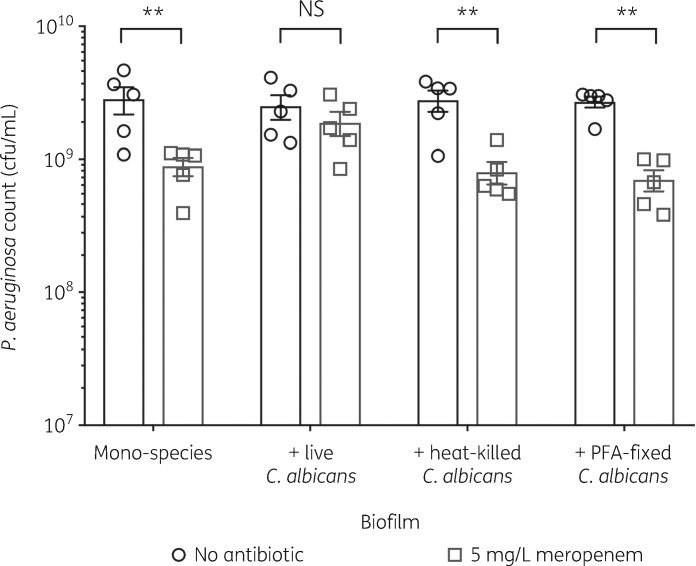
Increased tolerance to meropenem is dependent on fungal viability. Preformed 24 h biofilms were incubated for 18 h in MHB containing no antibiotic or 5 mg/L meropenem. Data are the mean ± SEM from five biological replicates. Data were analysed using two-way ANOVA and Holm–Sidak’s multiple comparisons test (**P <0.01). NS, not significant.

Production of biofilm ECM is an active process, involving secretion of glycoproteins, polysaccharides, lipids and nucleic acids.^2^ To determine whether the protective effect of *C. albicans* is mediated by an active or passive mechanism, *P. aeruginosa* biofilms were grown in the presence of either heat-killed *C. albicans* (disrupts the fungal cell wall, denatures proteins and causes cell lysis) or by fixing the *C. albicans* cells in 4% PFA (maintains cell structure). However, in the presence of heat-killed or PFA-fixed *C. albicans*, *P. aeruginosa* remained susceptible to meropenem (Figure 2), suggesting that *C. albicans* actively protects *P. aeruginosa* from meropenem.

### Candida dubliniensis also enhances P. aeruginosa meropenem tolerance in dual-species biofilms

**Figure 3.**
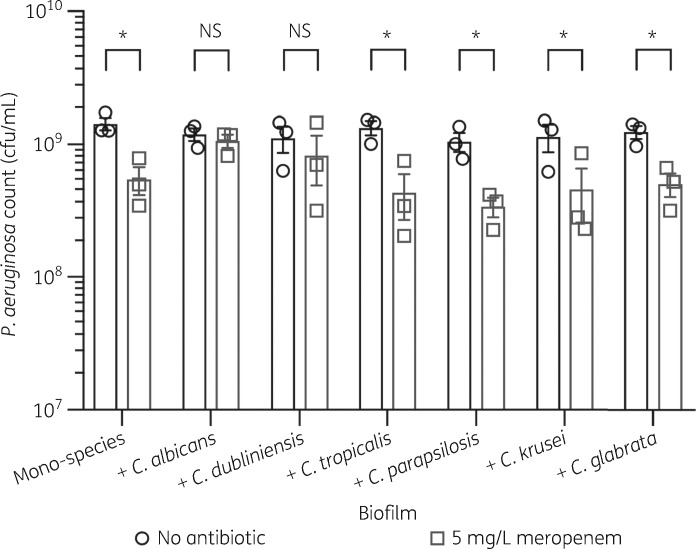
*C. dubliniensis* also enhances tolerance of *P. aeruginosa* to meropenem in dual-species biofilms. Preformed 24 h biofilms were incubated for 18 h in MHB containing no antibiotic or 5 mg/L meropenem. Data are the mean ± SEM from three biological replicates. Data were analysed using two-way ANOVA and Holm–Sidak’s multiple comparisons test (*P <* *0.05). NS, not significant.

Only a few species of the *Candida* genus are associated with disease in humans, including *C. albicans*, *C. dubliniensis*, *Candida tropicalis*, *Candida parapsilosis*, *Candida krusei* and *Candida glabrata*.^38^ To determine whether other *Candida* species can also protect *P. aeruginosa* from meropenem, biofilms were grown in the presence of these clinically relevant non-*albicans Candida* (NAC) species. Of the NAC species tested, only *C. dubliniensis* increased the tolerance of *P. aeruginosa* to meropenem (Figure 3). *C. dubliniensis* is phylogenetically most closely related to *C. albicans*,^39^ indicating that *C. albicans* and *C. dubliniensis* may enhance *P. aeruginosa* meropenem tolerance in a similar manner.

### Fungal cell wall polysaccharides enhance P. aeruginosa tolerance to meropenem

**Figure 4.**
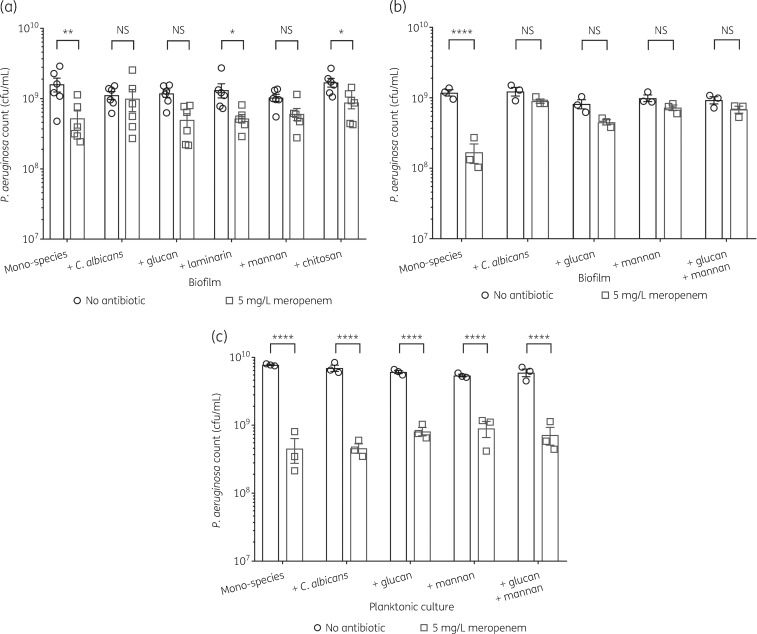
Mannan and glucan enhance *P. aeruginosa* biofilm tolerance to meropenem. Fungal polysaccharides were used at a final concentration of 0.25 mg/mL. (a) Mannan and glucan enhance *P. aeruginosa* biofilm tolerance to 5 mg/L meropenem. Preformed 24 h biofilms were incubated for 18 h in MHB containing no antibiotic or 5 mg/L meropenem. Data are the mean ± SEM from six biological replicates. (b) The effects of mannan and glucan are not additive. (c) Enhanced meropenem tolerance from mannan and glucan is biofilm specific. Planktonic cultures supplemented with exogenous mannan and/or glucan were grown for 3 h and subsequently incubated for 18 h in MHB containing 5 mg/L meropenem. Data are the mean ± SEM from three biological replicates. Data were analysed using two-way ANOVA and Holm–Sidak’s multiple comparisons test (*P < 0.05, **P < 0.01 and ****P < 0.0001). NS, not significant.

The secretion of ECM polymers, specifically polysaccharides, by *C. albicans* biofilm cells is linked to increased antifungal resistance of fungal biofilms.^40^^,^^41^ However, there is increasing evidence that fungal ECM polysaccharides also contribute to antibiotic resistance in dual-species fungal/bacterial biofilms.^34^^,^^42^^,^^43^ To determine whether secreted fungal cell wall polymers play a role in protecting *P. aeruginosa*, mono-species *P. aeruginosa* biofilms were grown in the presence of purified fungal cell wall polysaccharides, including glucan (a mix of β-1,3-glucan and β-1,6-glucan), laminarin (an isoform of β-1,3-glucan), mannan and chitosan (deacetylated chitin). Both mannan and glucan enhanced tolerance of *P. aeruginosa* to meropenem in biofilms (Figure 4a), but not in planktonic cultures (Figure 4c). To determine whether mannan and glucan have independent effects, *P. aeruginosa* biofilms were supplemented with glucan and mannan in combination. However, no additive effect was observed (Figure 4b). Mannan and glucan protected *P. aeruginosa* when added to mature biofilms at the same time as meropenem (Figure S4), suggesting that the polysaccharides may sequester or inhibit the activity of the drug. Therefore, *C. albicans* actively secretes mannan and/or glucan into the biofilm ECM, which protects *P. aeruginosa* from meropenem.

### C. albicans cell wall glycosylation is important for protection against meropenem

**Figure 5.**
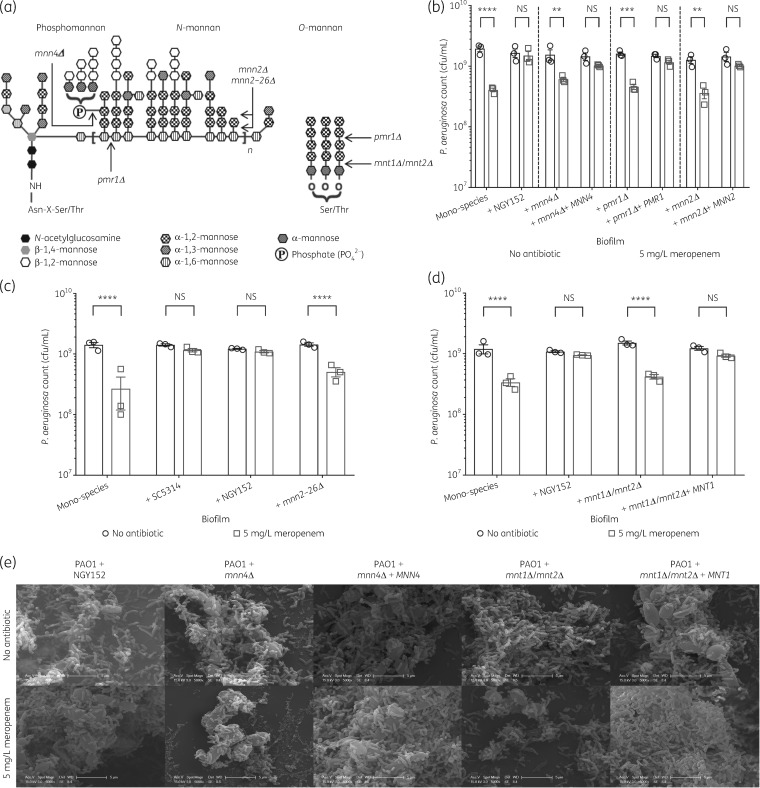
*C. albicans* cell wall glycosylation is important for protection against meropenem. (a) Schematic diagram representing the structure of *N*-mannan (including phosphomannan) and *O*-mannan of *C. albicans*.^44^^,^^46^ The points of truncation of the mutants used in this study are indicated by arrows. The *pmr1Δ* mutant causes loss of a Golgi Ca^2+^/Mn^2+^-ATPase, affecting numerous mannosyltransferases, so the extent of truncation of the α-1,6-mannose backbone is variable.^44^^,^^45^ (b) *N*-mannan glycosylation is important for protection against meropenem. Preformed 24 h biofilms were incubated for 18 h in MHB containing no antibiotic or 5 mg/L meropenem. The *N*-mannan glycosylation mutants (*mnn4Δ*, *pmr1Δ* or *mnn2Δ*) inhibit the ability of *C. albicans* to protect *P. aeruginosa*. Tolerance to meropenem is restored in reconstituted control strains. (c) The *mnn2–26Δ* sextuple mutant, in which only the unsubstituted α-1,6-mannose backbone of *N*-mannan remains, inhibits the ability of *C. albicans* to protect *P. aeruginosa*. (d) *O*-mannan glycosylation is important for protection against meropenem. The *mnt1Δ*/*mnt2Δ* double mutant inhibits the ability of *C. albicans* to protect *P. aeruginosa*. Meropenem tolerance is restored when *MNT1* is reconstituted. Data are the mean ± SEM from three biological replicates. Data were analysed using two-way ANOVA and Holm–Sidak’s multiple comparisons test (**P < 0.01, ***P < 0.001 and ****P <* *0.0001 in panels b, c and d). NS, not significant. (e) Scanning electron microscopy analysis of biofilms. Deletion of genes required for fungal *N*-mannan biosynthesis (*mnn4*) or *O*-mannan biosynthesis (*mnt1*/*mnt2*) reduced the ability of *C. albicans* to protect *P. aeruginosa* from meropenem, as indicated by the reduction in the number of bacterial cells following meropenem treatment; the majority of surviving bacteria are in close contact with fungal cells. When the genes (*MNN4* or *MNT1*) are reconstituted, the protective effect is restored, as evidenced by the abundance of *P. aeruginosa* cells coating the fungi in the meropenem-treated samples.

To further investigate the role of fungal mannan in meropenem tolerance, the ability of *C. albicans* cell wall glycosylation mutants to protect *P. aeruginosa* was quantified. *C. albicans* has two major forms of mannan, the extensively branched N-linked mannan and the simple, linear O-linked mannan. Deletion of genes involved in key glycosylation steps results in the incorporation of altered mannan epitopes in the cell wall (Figure 5a) and within the ECM.^44–46^ To elucidate the role of these glycosylation structures in protecting *P. aeruginosa* from meropenem, mutants defective in general protein glycosylation (*pmr1Δ*, ATPase required for transporting Ca^2+^ and Mn^2+^ ions into the Golgi), *N*-mannan phosphomannan incorporation (*mnn4Δ*), *N*-mannan side chain elaboration (*mnn2Δ*, *mnn2–26Δ*) and *O*-mannan biosynthesis (*mnt1Δ/mnt2Δ*) were tested for their ability to protect *P. aeruginosa* from meropenem. Deletion of genes required for *N*-mannan biosynthesis (*mnn4*, *mnn2–26* and *pmr1*) reduced the ability of *C. albicans* to protect *P. aeruginosa* from meropenem (Figure 5b and c). Scanning electron microscopy analysis showed very few bacterial cells surviving meropenem treatment in dual-species biofilms with *C. albicans* glycosylation mutants (Figure 5e) but bacteria that did survive were closely adhered to the fungal cell surface, suggesting cell–cell adherence may play a role in meropenem tolerance. In agreement with this, deletion of genes involved in *O*-mannan biosynthesis (*mnt1*/*mnt2*), which have previously been shown to be involved in bacterial attachment to *C. albicans*,^47^ had the greatest impact on *P. aeruginosa* protection (Figure 5d and e). This indicates that protection of *P. aeruginosa* requires full elaboration of fungal mannan.

### C. albicans does not enhance P. aeruginosa tolerance to other antibiotics

To determine whether the presence of *C. albicans* also affects tolerance of *P. aeruginosa* biofilm cells to other clinically relevant antibiotics, mono- and dual-species biofilms were treated with 5 mg/L ceftazidime, 0.05 mg/L ciprofloxacin, 2 mg/L tobramycin or a combination of 5 mg/L meropenem and 2 mg/L tobramycin. However, the presence of *C. albicans* did not provide protection against these antimicrobial treatments (Figure S5a and b), suggesting that the mechanism by which *C. albicans* confers enhanced tolerance is likely due to the chemical structure of meropenem.

### C. albicans protects P. aeruginosa CF isolates from meropenem

**Figure 6.**
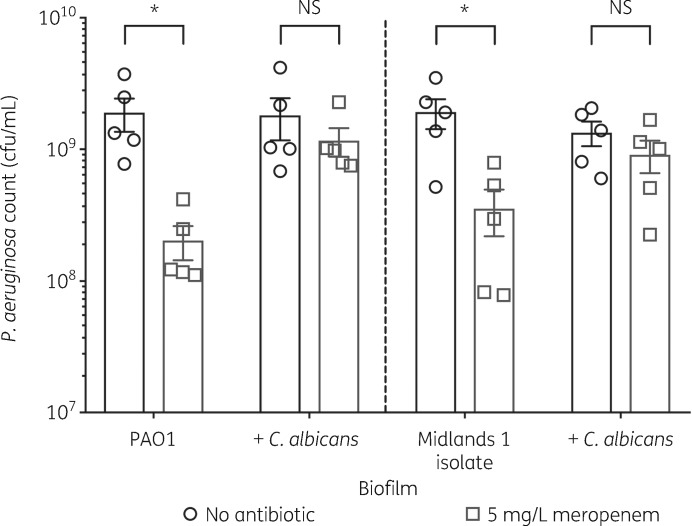
*C. albicans* enhances meropenem tolerance of a *P. aeruginosa* CF isolate. Preformed 24 h biofilms were incubated for 18 h in MHB containing no antibiotic or 5 mg/L meropenem. Data are the mean ± SEM from five biological replicates. Data were analysed using two-way ANOVA and Holm–Sidak’s multiple comparisons test (*P < 0.05). NS, not significant.

To explore the clinical relevance of the above findings, the ability of *C. albicans* to increase meropenem tolerance of clinical CF isolates was tested. Mono-species biofilms of the Midlands 1 CF isolate^48^ were susceptible to meropenem. However, *P. aeruginosa* survival was unaffected during growth in a dual-species biofilm with *C. albicans* (Figure 6), suggesting that in CF patients, co-colonization with *C. albicans* may increase *P. aeruginosa* tolerance to meropenem.

## Discussion

*P. aeruginosa* and *C. albicans* are commonly co-isolated from the sputum of CF patients,^20^ where the thickened mucus layer lining the endothelium of the lower respiratory tract provides an ideal environment for biofilm formation.^3^^,^^7^ Chronic *P. aeruginosa* colonization in the CF lung is correlated with increased likelihood of *C. albicans* colonization, indicating a synergistic interaction that leads to a greater decline in lung function.^10^^,^^11^ Here, we show that *C. albicans* significantly enhanced *P. aeruginosa* biofilm tolerance to 5 mg/L meropenem in both a laboratory strain and a CF isolate. Although the protective effect was relatively small (<1 log_10_ change in *P. aeruginosa* cfu/mL), this may still have clinical relevance. For example, the clinical breakpoint of *P. aeruginosa* for meropenem is >8 mg/L;^32^ this increased tolerance could push the required meropenem concentration over the clinical breakpoint, categorizing the infection as meropenem resistant. This has direct implications for treatment of CF patients who present with coinfection. Standard doses of meropenem may become insufficient for treating *P. aeruginosa* infection, with combination antibiotic/antifungal therapy being a potentially more effective therapeutic option.

The protective effect of *C. albicans* was biofilm specific and dependent upon fungal ECM components. This is consistent with other reports where *C. albicans* ECM components have been shown to provide protection against ofloxacin and vancomycin in dual-species biofilms with *Escherichia coli* and *Staphylococcus aureus*, respectively.^42^^,^^43^ However, in contrast to these studies, where *C. albicans* ECM components provide protection against a range of antimicrobials, *C. albicans* ECM components only increased *P. aeruginosa* tolerance to meropenem. This suggests that the mechanism by which *C. albicans* ECM components enhance meropenem tolerance may be different to those proposed for other antibiotics. Considering the effect was not seen for other β-lactams (i.e. ceftazidime) this suggests the protective mechanism may depend on chemical structure or ability to bind mannan or β-glucan, rather than the mode of action.

Previously, mannan and β-glucan have been shown to bind and sequester antimicrobials, limiting their diffusion through biofilms.^43^ Therefore, it is possible that the actual concentration of meropenem within dual-species biofilms is significantly lower. Similar interactions have been observed in dual-species biofilms where *Streptococcus mutans* exopolysaccharides bind and sequester fluconazole, reducing its efficacy against *C. albicans*.^49^ Alternatively, mannans or glucans may coat bacterial cells, providing a physical barrier that impedes drug permeation, supporting the proposed mechanism by which *S. aureus* is protected from vancomycin.^42^

Although *C. albicans* remains clinically the most commonly isolated *Candida* species, the prevalence of NAC species is increasing.^50^*C. dubliniensis* was the only NAC species that protected *P. aeruginosa* from meropenem. This finding is of clinical relevance as, although less common than *C. albicans*, the prevalence of *C. dubliniensis* within CF patients ranges from 2.6% to 39.0% and there are cases of *C. dubliniensis* being co-isolated with *P. aeruginosa* from the lower respiratory tracts of CF patients.^51^*C. dubliniensis* is the most closely related NAC species to *C. albicans* and, as a result, their biofilms are structurally similar,^38^^,^^39^ with networks of yeast and hyphal cells embedded in a comparable ECM.^52^ Although the other NAC species produce biofilms, the composition of their ECM is considerably different^53^^,^^54^ and their biofilms lack hyphae, which are important for bacterial attachment. Scanning electron microscopy confirmed that most bacteria in the treated dual-species biofilms were attached to fungal hyphae, suggesting that this interaction is important for protection. This hypothesis is supported by the fact that removal of *O*-mannan, which is required for bacterial binding,^47^^,^^55^ reduced the ability of *C. albicans* to protect *P. aeruginosa*. However, given that purified carbohydrates were able to provide similar protection to *C. albicans*, it would suggest that ECM composition is the major contributing factor providing antimicrobial protection.

In conclusion, secreted *C. albicans* ECM polysaccharides protect *P. aeruginosa* by reducing the efficacy of meropenem. Clinically, this could result in persistent bacterial infection due to pockets of protected cells, which may then acquire true resistance as a result of continued exposure to subMIC concentrations of antibiotics. This highlights the importance of early diagnosis of dual-species biofilm infections, so that more efficacious therapeutic options, such as combination antibiotic/antifungal therapy, can be considered.

## Supplementary Material

dkz514_Supplementary_Data
