# Supplementary material for: Candida albicans enhances meropenem tolerance of Pseudomonas aeruginosa in a dual-species biofilm
Source: J Antimicrob Chemother. 2019 Dec 22;75(4):925–35. doi: 10.1093/jac/dkz514 (PMC7069478; doi:10.1093/jac/dkz514)
Supplement: dkz514_Supplementary_Data [file dkz514_supplementary_data.docx]

**Supplementary data**

**
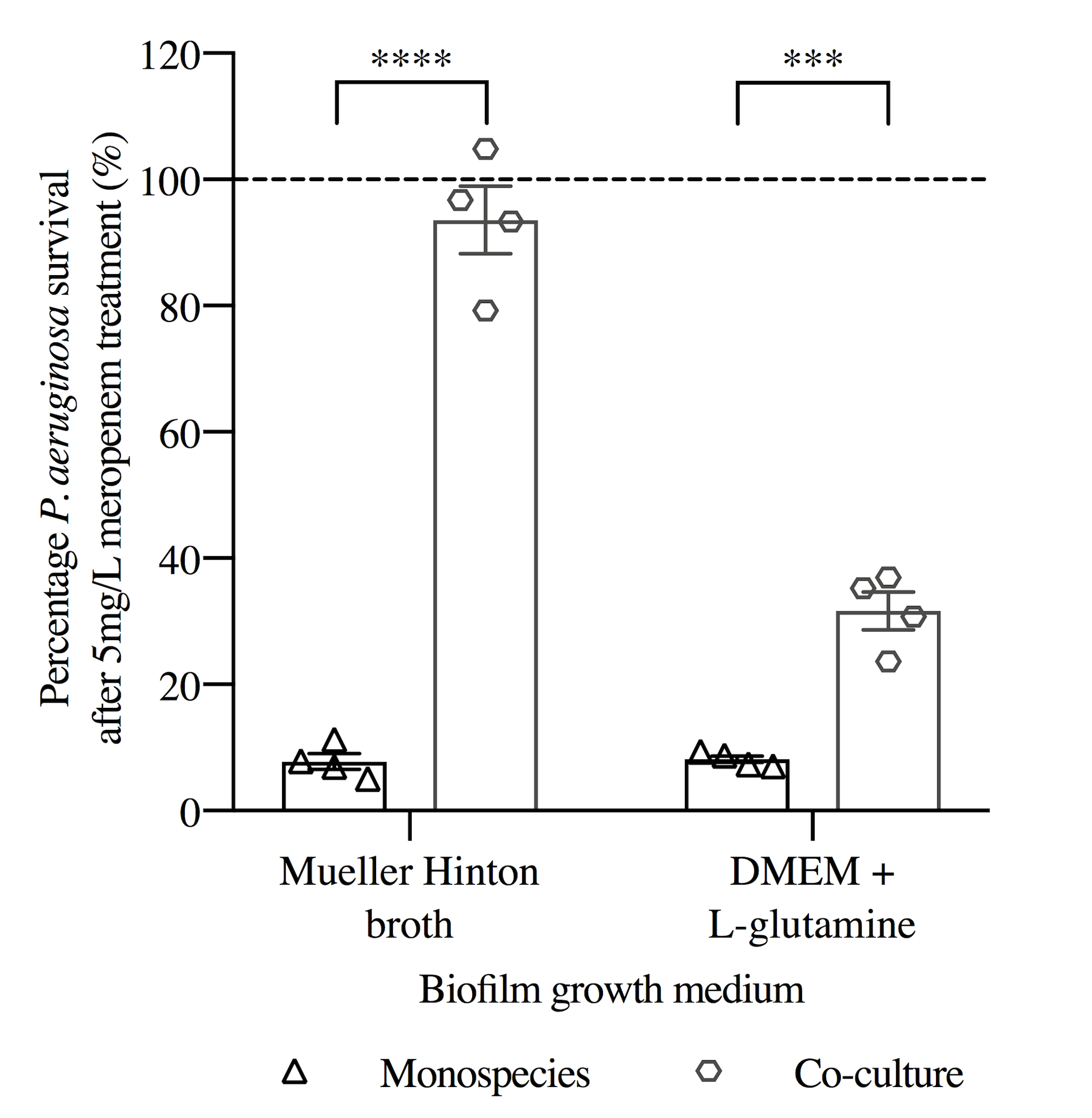
**

**Figure S1. *C. albicans* increases the tolerance of *P. aeruginosa* to meropenem in both Mueller Hinton broth and DMEM.** Preformed 24 h biofilms were incubated in DMEM (supplemented with 1% L-glutamine) or Mueller Hinton broth containing no antibiotic or 5 mg/L meropenem, for 18 h. Data show the mean ± the SEM from 4 biological replicates. Data were analysed using 2-way ANOVA and Holm-Sidak’s multiple comparisons test (*** P < 0.001; **** P < 0.0001).

**
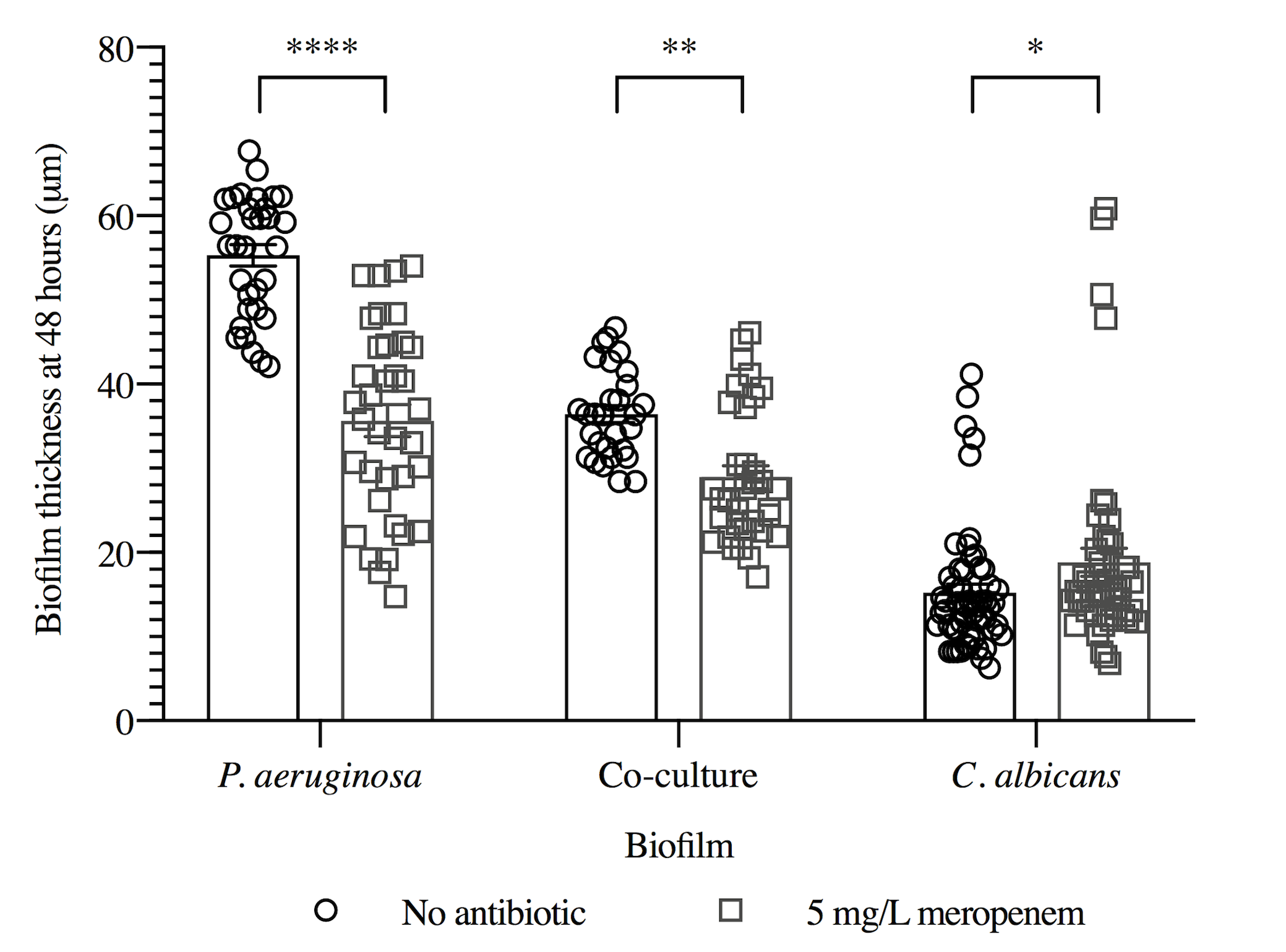
**

**Figure S2. Quantification of biofilm thickness from confocal microscopy images.** Preformed 24 h biofilms were incubated in Mueller Hinton broth containing 0 or 5 mg/L meropenem, for 18 h. Biofilms were fixed, stained and washed, before confocal images were taken from 2–3 areas per biofilm. Data show the mean ± the SEM from 2 biological replicates. Data were analysed using 2-way ANOVA and Holm-Sidak’s multiple comparisons test (* P < 0.05; ** P < 0.01; **** P < 0.0001).

**
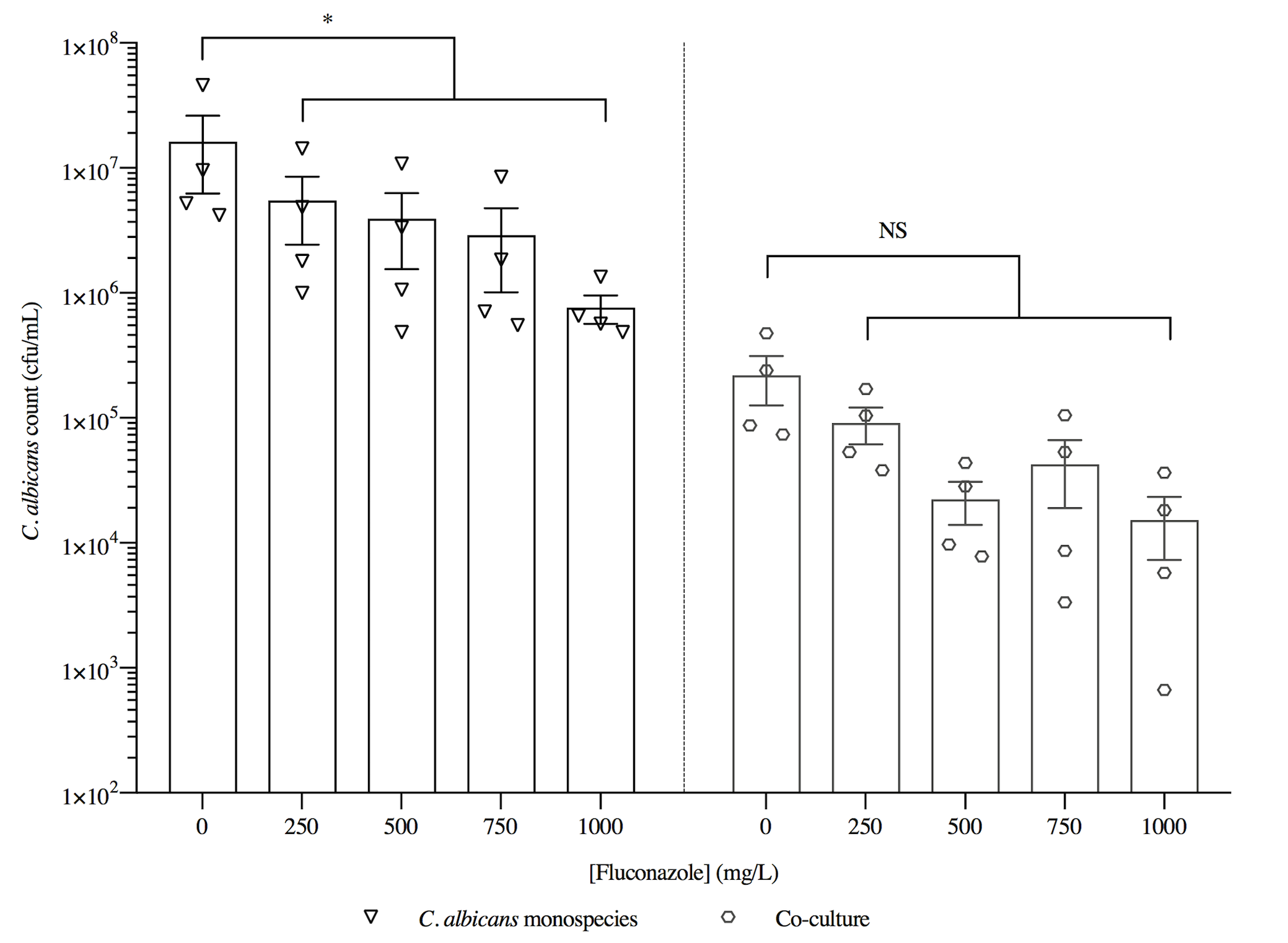
**

**Figure S3. *P. aeruginosa* does not increase *C. albicans* sensitivity to fluconazole.** Preformed 24 h biofilms were incubated in Mueller Hinton broth containing 0, 250, 500, 750 or 1000 mg/L fluconazole for 18 h. Data are the mean ± the SEM from 4 biological replicates. Data were analysed using 2-way ANOVA and Holm-Sidak’s multiple comparisons test (* P < 0.05).

**
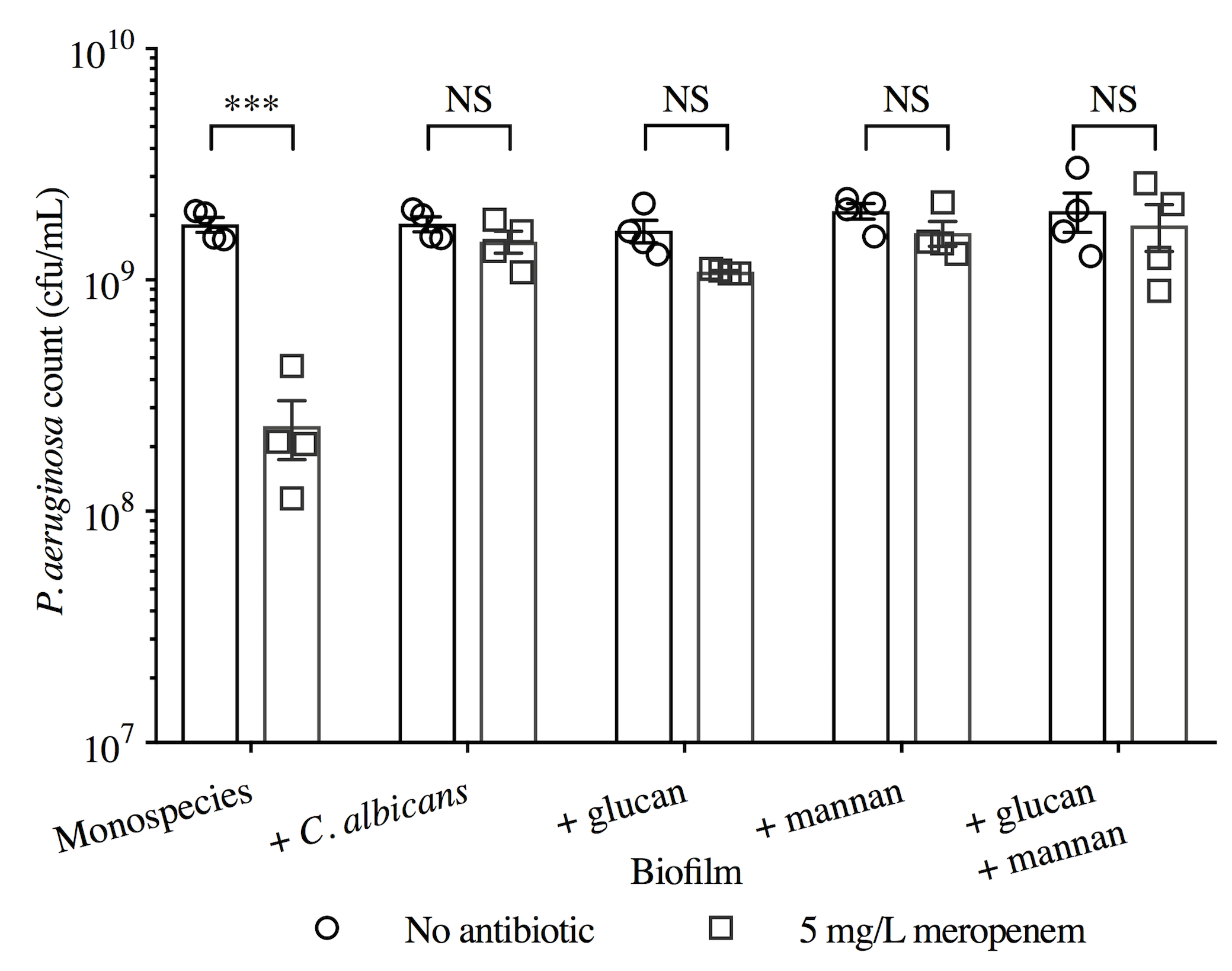
**

**Figure S4. Mannan and glucan enhance *P. aeruginosa* biofilm tolerance to meropenem, even when added to pre-formed biofilms.** Pre-formed 24 h biofilms were incubated for 18 h in Mueller Hinton broth containing no antibiotic, 5 mg/L meropenem, 0.25 mg/mL mannan or glucan, or a combination of these. Data are the mean ± the SEM from 4 biological replicates. Data were analysed using 2-way ANOVA and Holm-Sidak’s multiple comparisons test (*** P < 0.001).

**
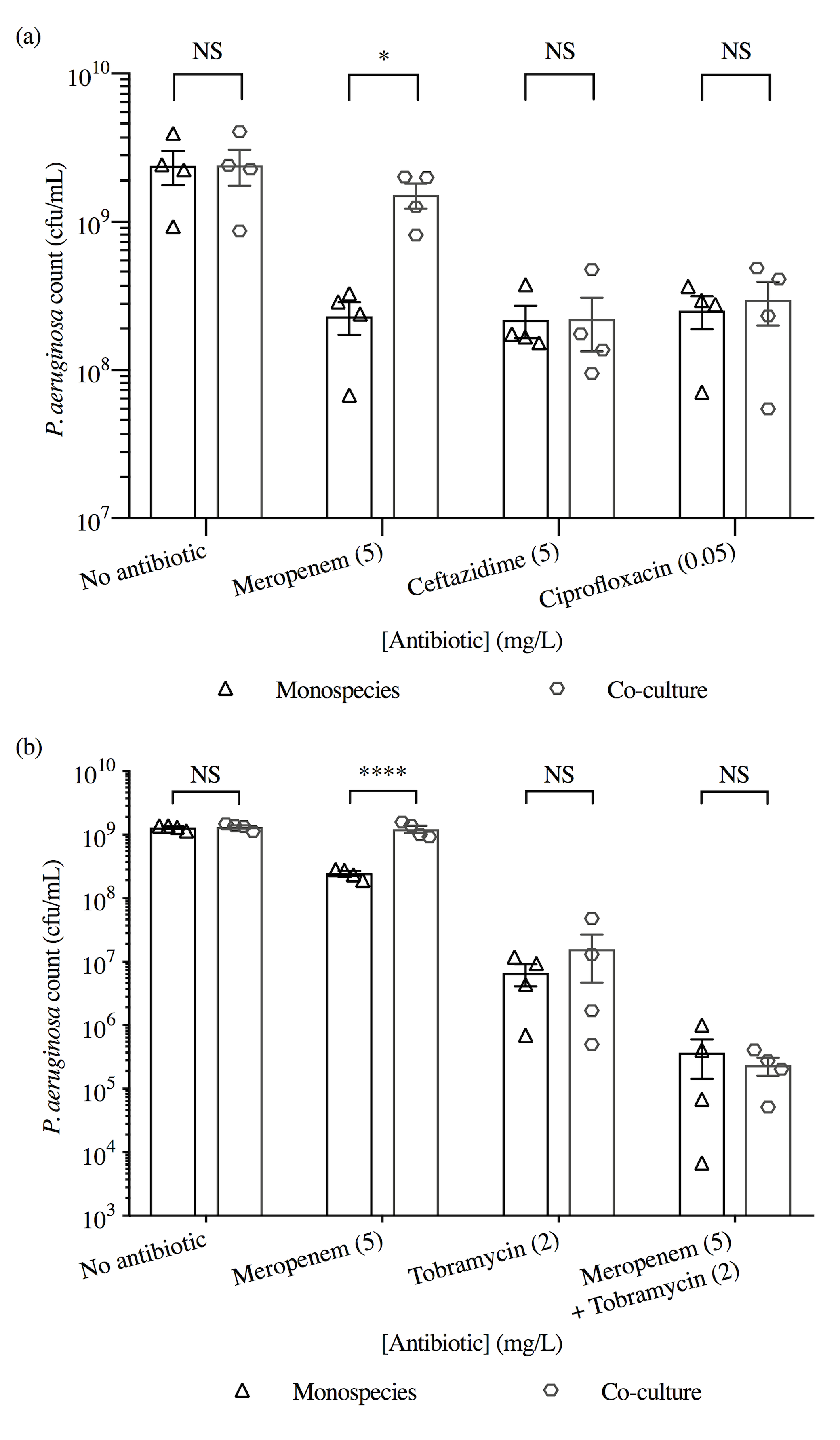
**

**Figure S5. *C. albicans* does not enhance *P. aeruginosa* tolerance to other antibiotics.** Preformed 24 h biofilms were incubated for 18 h in Mueller Hinton broth containing meropenem (5 mg/L), ceftazidime (5 mg/L), ciprofloxacin (0.05 mg/L), tobramycin (2 mg/L), or a combination of meropenem and tobramycin. These antibiotics were chosen due to their clinical relevance: meropenem is combined with the aminoglycoside, tobramycin, for the treatment of *P. aeruginosa* infections in the CF lung;^1^ the cephalosporin, ceftazidime, is a first-line therapy for the treatment of *P. aeruginosa* infections; and the fluoroquinolone, ciprofloxacin, is used to treat chronic *P. aeruginosa* infections in the CF lung.^2^ The concentrations tested represent those found in patients; the concentration of ceftazidime found in the epithelial lining fluid following a standard dose (20 mg per kg of body weight) is 6 mg/L,^3^ the concentration of ciprofloxacin found in the epithelial lining fluid 24 h after a 500mg dose is negligible,^4^ and that of tobramycin 2 h after treatment is 1.67 mg/L.^5^ **(a)** *C. albicans* does not enhance *p. aeruginosa* tolerance to ceftazidime or ciprofloxacin. **(b)** *C. albicans* does not enhance *p. aeruginosa* tolerance to tobramycin or combination meropenem–tobramycin treatment. Data are the mean ± the SEM from 4 biological replicates. Data were analysed using 2-way ANOVA and Holm-Sidak’s multiple comparisons test (* P < 0.05; **** P < 0.0001).

**References**

**1** Cystic Fibrosis Trust. Antibiotic treatment for cystic fibrosis: report of the UK Cystic Fibrosis Trust antibiotic working group. 3rd Ed. 2009; <https://www.cysticfibrosis.org.uk/~/media/documents/life-with-cf/care-and-treatment/cd_antibiotic_treatment_for_cf_may_09.ashx?la=en>.

**2** Wagner S, Sommer R, Hinsberger S *et al.* Novel strategies for the treatment of *Pseudomonas aeruginosa* infections. *J Med Chem* 2016; **59**: 5929-69.

**3** Cousson J, Floch T, Guillard T *et al.* Lung concentrations of ceftazidime administered by continuous versus intermittent infusion in patients with ventilator-associated pneumonia. *Antimicrob Agents Chemother* 2015; **59**: 1905-9.

**4** Gotfried MH, Danziger LH, Rodvold KA. Steady-state plasma and intrapulmonary concentrations of levofloxacin and ciprofloxacin in healthy adult subjects. *Chest* 2001; **119**: 1114-22.

**5** Carcas AJ, García-Satué JL, Zapater P *et al.* Tobramycin penetration into epithelial lining fluid of patients with pneumonia. *Clin Pharmacol Ther* 1999; **65**: 245-50.
